# Comparison of the Outcomes of Lateral Wall, Perimodiolar, and Midscala Electrodes in Cochlear Implantation Surgery: A Meta-Analysis

**DOI:** 10.1055/s-0046-1820087

**Published:** 2026-10-14

**Authors:** Samer Ahmed Ibrahim, Tamer Shoukry Sobhy, Sara Ashraf Shoeib, Ahmed Abdelmoneim Teaima

**Affiliations:** 1 Department of Otorhinolaryngology, Faculty of Medicine, Ain Shams University, Cairo, Egypt

**Keywords:** cochlear cmplantation, perimodiolar electrodes, midscala electrode, residual hearing

## Abstract

**Introduction:**

Cochlear implant (CI) is the standard of care for most cases of severe-to-profound hearing loss. Different electrodes are available for CI.

**Objective:**

To compare the lateral wall, midscala, and perimodiolar electrodes in CI surgery in terms of trauma to the cochlea and hearing preservation (if there is residual hearing).

**Data Synthesis:**

We performed a comprehensive search on the PubMed/MEDLINE, Scopus, Web of Science, and Cochrane Library databases in June 2022 for articles in the literature published until this year. We included studies published in English, involving human subjects, and regarding the types of electrodes in CI surgery, and we excluded laboratory and opinion studies. The current review followed the guidelines of the 2009 Preferred Reporting Items for Systematic Reviews and Meta-analyses (PRISMA) statement. We found 21 studies; the hearing preservation rates with each electrode were as follows: lateral wall electrode – 53%; perimodiolar electrode – 35%; and midscala electrode – 58%. The lateral wall and midscala electrodes presented better preservation of residual hearing than the perimodiolar electrode. The rates of cochlear trauma with each electrode were as follows: lateral wall electrode – 61%; perimodiolar electrode – 29%; and midscala electrode – 16%.

**Conclusion:**

The rates of preservation of functional residual hearing in CI are higher in lateral wall and midscala electrodes than in perimodiolar electrodes.

## Introduction

Cochlear implant (CI) is the most successful neuroprosthetic device and the standard of care for most cases of severe-to-profound hearing loss. 1 Due to marked improvement in word recognition scores after CI, its indications have been expanded to patients with residual hearing. Moreover, preoperative residual hearing is a positive predictor of better performance after CI. Therefore. atraumatic surgery becomes essential to preserve residual hearing and enhance electroacoustic stimulation (EAS) after the procedure. 2 3 The type of electrode is very important to achieve the best result in terms of EAS and avoid trauma to the basilar membrane, lateral wall or osseous spiral lamina. 4 Lateral wall (straight) electrodes are designed to reside at the lateral wall of the cochlea, while perimodiolar electrodes are for closer position, in relation to the modiolus, and midscala electrodes are precurved arrays inserted between the lateral wall and the modiolus. 5 There are conflicting data in the literature to reinforce the advantages and drawbacks related to of each electrode. Thus, we have conducted the present study to compare the lateral wall, midscala, and perimodiolar electrodes regarding trauma to the cochlea and hearing preservation (if there is residual hearing).

## Methods

### Literature Review

#### Literature Search

We conducted a comprehensive search on the PubMed/MEDLINE, Scopus, Web of Science, and Cochrane Library databases for articles published in English on CI surgery using a combination of the following keywords: *lateral wall electrode* , *midscala electrode* , *perimodiolar electrode* , *cochlear implant* , *hearing preservation* , *outcome* , and *cochlear trauma* . The article selection and screening strategy was based on the 2009 Preferred Reporting Items for Systematic Reviews and Meta-analyses statement ( Fig. 1 ). The references of the screened articles were also assessed for relevance to maximize sensitivity. The search yielded 186 articles, 77 of which 77 met the inclusion criteria. After duplicate removal, the titles and abstracts of 67 articles were screened, and 21 articles 2 6 7 8 9 10 11 12 13 14 15 16 17 18 19 20 21 22 23 24 25 were included after full-text reading. Detailed characteristics of the included studies are shown in Table 1 .

**Fig. 1:**
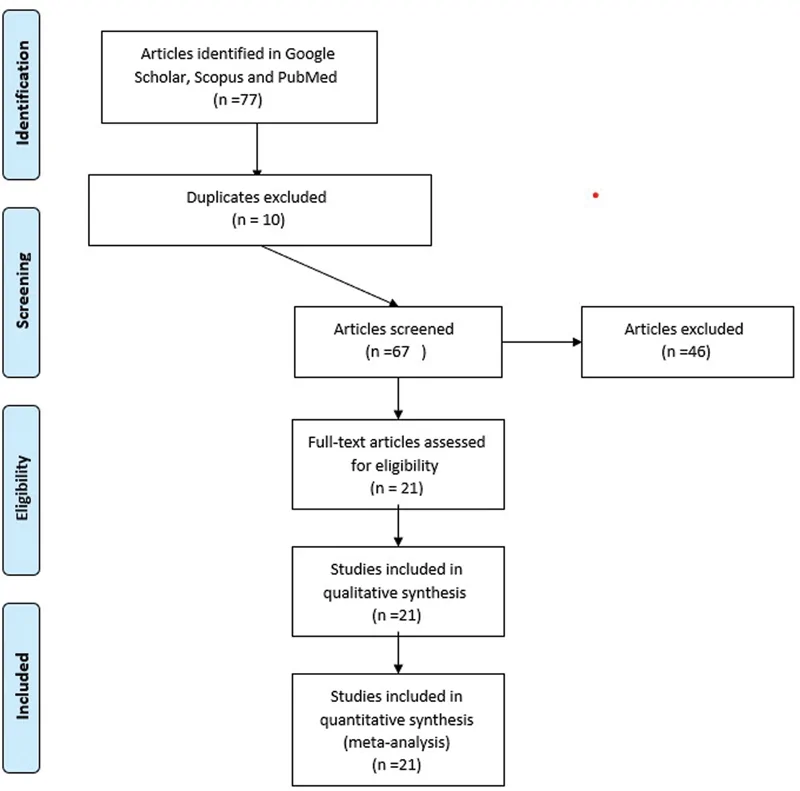
Preferred Reporting Items for Systematic Reviews and Meta-analyses (PRISMA) flow diagram of the search and review process.

**Table 1:** Characteristics table for patient in the included articles

Author, year (country)	Type of study	Sample size	Type of electrode used
Aschendroff et al. 6 (2017), Germany	Prospective study	45	Perimodilar electrodes
Berreittini et al. 7 (2008), Italy	Retrospective study	30	19 Lateral wall electrodes; 11 perimodiolar electrodes
Boyer et al. 2 (2015), France	Prospective study	61	30 lateral wall electrodes; 31 perimodiolar electrodes
Dees et al. 8 (2018), Switzerland	Prospective study	14	14 Midscala electrodes
Durakovic et al. 9 (2020), United States	Case series study	76	Perimodilar electrodes
Fabie et al. 10 (2018), United States	Retrospective study	328	234 lateral wall electrodes; 46 perimodiolar electrodes; 48 midscala electrodes
Friedmann et al. 11 (2019), United States	Retrospective study	237	Perimodiolar electrodes
Harrison et al. 12 (2020), United Kingdom	Retrospective study	53	Lateral wall electrodes
Hunter et al. 13 (2016), United States	Retrospective study	50	Midscala electrodes
Lenarz et al. 14 (2020), Germany	Prospective study	20	Lateral wall electrodes
Liebscher et al. 15 (2021), Germany	Retrospective study	255	Perimodiolar electrodes
Mady et al. 16 (2017), United States	Case series study	45	21 lateral wall electrodes; 24 perimodiolar electrodes
Schart-Morén et al. 17 (2020), Sweden	Retrospective study	15	Lateral wall electrodes
Selleck et al. 8 (2019), United States	Retrospective study	105	71 lateral wall electrodes; 33 perimodiolar electrodes 1 Midscala electrode
Sipari et al. 19 (2018), Finland	Retrospective case study	31	Midscala electrodes
Sodamerhy et al. 20 (2008), Mexico	Longitudinal, observational, comparative study	48	26 lateral wall electrodes; 22 perimodiolar electrodes
Svrakic et al. 21 (2016), United States	Retrospective study	126	63 lateral wall electrodes; 63 midscala electrodes
Todt et al. 22 (2016), Germany	Prospective study	20	Midscala electrodes
Wanna et al. 23 (2014), United States	Comparative longitudinal study	116	47 lateral wall electrodes; 69 perimodiolar electrodes
Wanna et al. 24 (2015), United States	Prospective study	45	27 lateral wall electrodes; 15 perimodiolar electrodes; 3 midscala electrodes
Wanna et al. 25 (2018), United States	Retrospective study	225	135 lateral wall electrodes; 55 perimodiolar electrodes; 35 midscala electrodes

#### Quality Assessment

The quality of the studies was assessed using the United States National Heart, Lung, and Blood Institute's study quality assessment tools ( https://www.nhlbi.nih.gov/health-topics/study-quality-assessment-tools ). The PRISMA checklist was used to evaluate the reporting quality of the included articles; the maximum score is 27 points, assigned as follow(Yes=1, No=0, NA=0).

### Statistical Analysis

The statistical analysis was performed using the MetaXL software, version 5.3 (EpiGear International Pty Ltd). Binary outcomes were presented as odds ratios (ORs) with 95%CIs. The incidence rates were presented as proportions with 95%CIs. Heterogeneity across studies was evaluated using the Cochrane Q test and the I-squared ( *I ^2^* ) statistic; values > 50% and/or a statistically significant Cochrane Q test ( *p* < 0.1) was regarded as significant heterogeneity.

Publication bias was assessed through an examination of funnel plots (effect size versus standard error) and Doi plots (effect size versus z-score) for asymmetry. The Luis Furuya-Kanamori (LFK) asymmetry index was used to quantify the degree of Doi plot asymmetry. Negative or positive LFK values < 1 denote no asymmetry, values ranging between 1 and 2 denote mild asymmetry, and values exceeding 2 denote major asymmetry.

## Results

In the single-arm meta-analysis, the rates of hearing preservation for each electrode were as follows: lateral wall – 53% ( Fig. 2 ); perimodiolar – 35% ( Fig. 3 ); and midscala – 58% ( Fig. 4 ); and the rates of cochlear trauma were as follows: lateral wall – 61% ( Fig. 5 ); perimodiolar – 29% ( Fig. 6 ); and midscala – 16% ( Fig. 7 ).

**Fig. 2:**
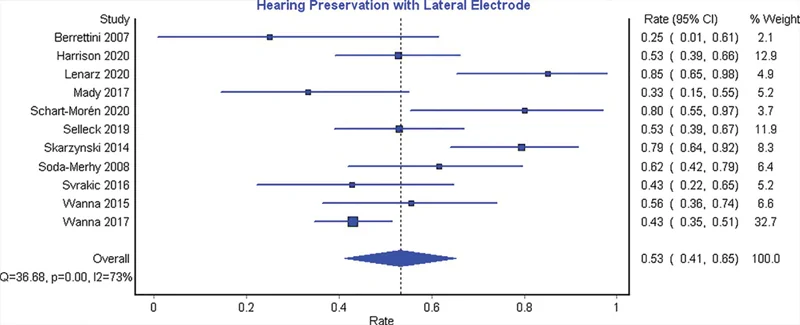
Forest plot for the rate of hearing preservation with lateral wall electrodes. Pooled rate (95%CI) = 53%. There is important heterogeneity across studies (Cochrane Q: *p* < 0.01; *I ^2^* = 73%).

**Fig. 3:**
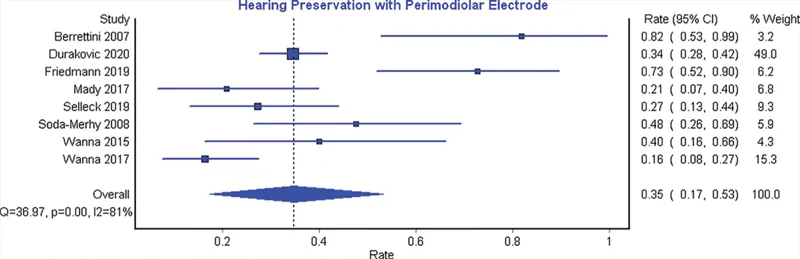
Forest plot for the rate of hearing preservation with perimodiolar electrodes. Pooled rate (95%CI) = 35%. There is important heterogeneity across studies (Cochrane Q: *p* < 0.01; *I ^2^* = 73%).

**Fig. 4:**
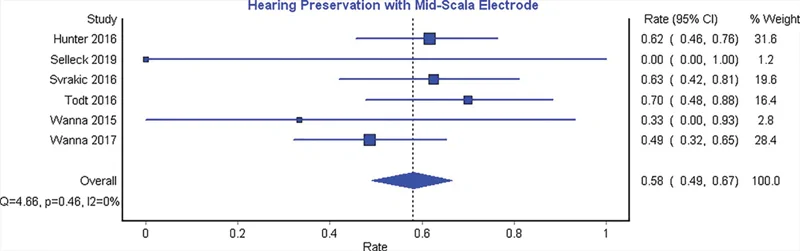
Forest plot for the rate of hearing preservation with midscala electrodes. Pooled rate (95%CI) = 58%. There is unimportant heterogeneity across studies (Cochrane Q: *p* = 0.46; *I ^2^* = 0%).

**Fig. 5:**
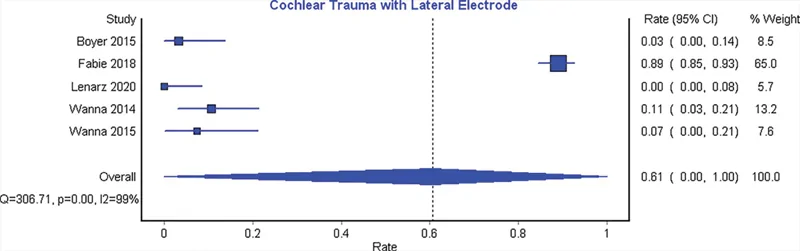
Forest plot for the rate of cochlear trauma with lateral wall electrodes. Pooled rate (95%CI) = 61%. There is important heterogeneity across studies (Cochrane Q: *p* < 0.01; *I ^2^* = 99%).

**Fig. 6:**
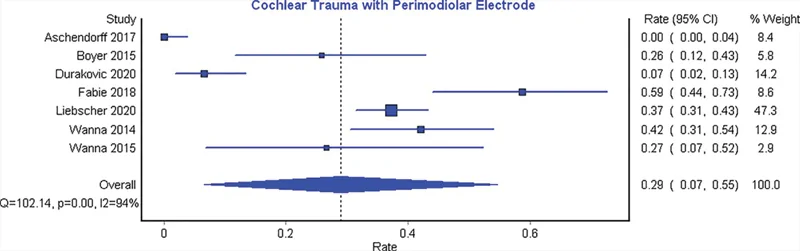
Forest plot for the rate of cochlear trauma with perimodiolar electrodes. Pooled rate (95%CI) = 29%. There is important heterogeneity across studies (Cochrane Q: *p* < 0.01; *I ^2^* = 99%).

**Fig. 7:**
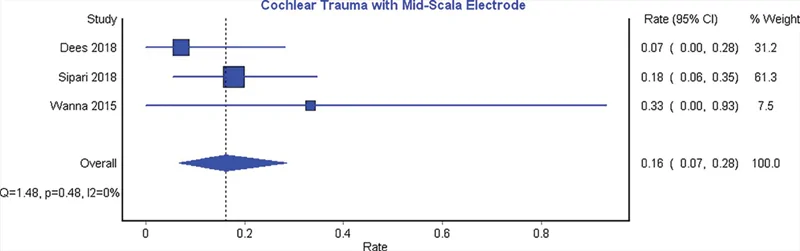
Forest plot for the rate of cochlear trauma with midscala electrodes. Pooled rate (95%CI) = 16%. There is important heterogeneity across studies (Cochrane Q: *p* = 0.48; *I ^2^* = 0%).

In the two-arm meta-analysis, the pooled OR (95%CI) for the comparison of the lateral wall and perimodiolar electrodes in terms of hearing preservation was of 2.20, favoring the lateral wall electrode ( Fig. 8 ); for the comparison of the lateral wall and midscala electrodes in terms of hearing preservation, it was of 0.79, favoring no electrode type over the other ( Fig. 9 ); and for the comparison of the periomodiolar and midscala electrodes in terms of hearing preservation, it was of was 0.30, favoring the midscala electrode ( Fig. 10 ). As for cochlear trauma, the pooled OR (95%CI) for the comparison of the lateral wall and perimodiolar electrodes was of 0.87, favoring no electrode over the other ( Fig. 11 ); for the comparison of the lateral wall and midscala electrodes, it was of 0.16, favoring no electrode over the other ( Fig. 12 ); and for the comparison of the perimodiolar and midscala electrodes, it was of 0.73, again favoring no electrode type over the other ( Fig. 13 ).

**Fig. 8:**
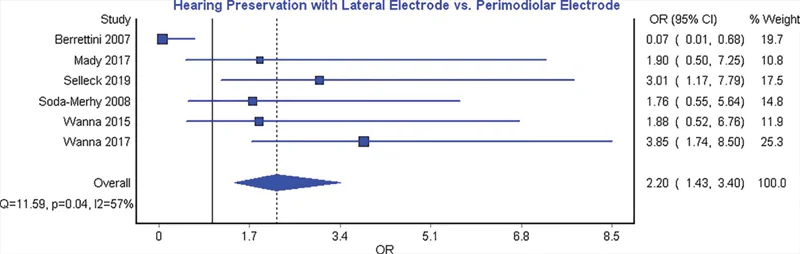
Forest plot for the comparison of the lateral wall and perimodiolar electrodes in terms of hearing preservation. Pooled odds ratio (95%CI) = 2.20 favoring Lateral electrode over Perimodiolar electrode. There is important heterogeneity across studies (Cochrane Q *p* = 0.04, I-squared = 57%).

**Fig. 9:**
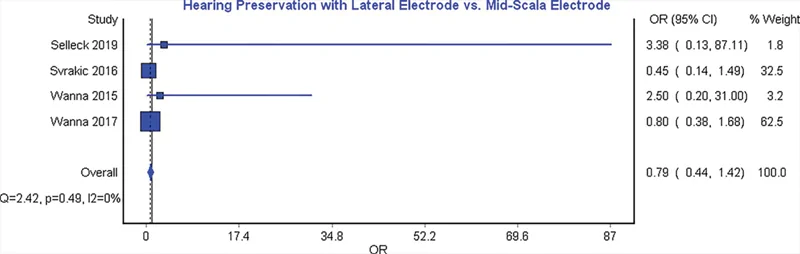
Forest plot for the comparison of the lateral wall and midscala electrodes in terms of hearing preservation. Pooled odds ratio (95%CI) = 0.79, favoring no electrode type over the other. There is important heterogeneity across studies (Cochrane Q: *p* = 0.04; *I ^2^* = 57%).

**Fig. 10:**
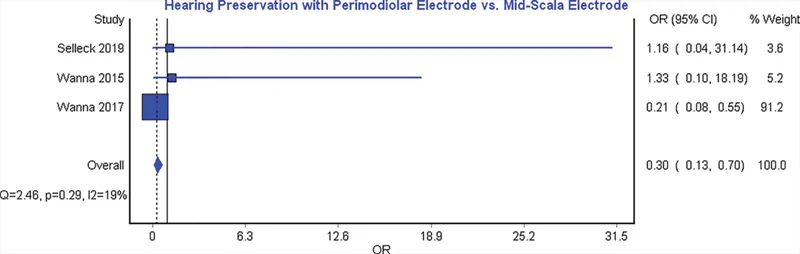
Forest plot for the comparison of the perimodiolar and midscala electrode s in terms of hearing preservation. Pooled odds ratio (95%CI) = 0.30, favoring the midscala electrode. There is important heterogeneity across studies (Cochrane Q: *p* = 0.04; *I ^2^* = 57%).

**Fig. 11:**
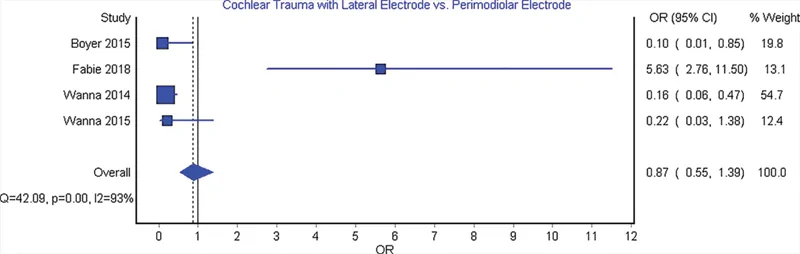
Forest plot for the comparison of the lateral wall and perimodiolar electrode s in terms of cochlear trauma. Pooled odds ratio (95%CI) = 0.87, favoring no electrode type over the other. There is important heterogeneity across studies (Cochrane Q: *p* < 0.01; *I ^2^* = 99%).

**Fig. 12:**
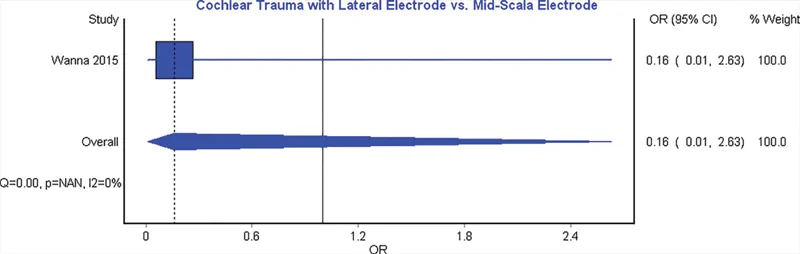
Forest plot for the comparison of the lateral wall and midscala electrodes in terms of cochlear trauma. Odds ratio (95%CI) = 0.16, favoring no electrode type over the other.

**Fig. 13:**
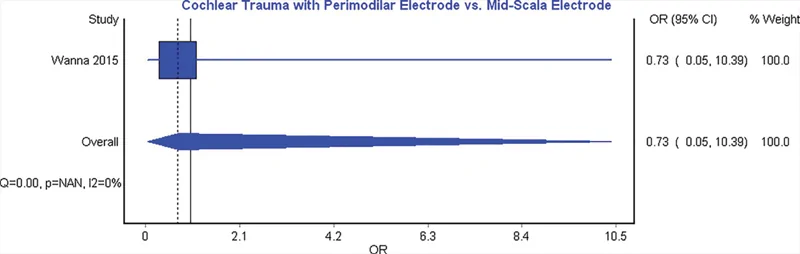
Forest plot for the comparison of the perimodilar and midscala electrodes in terms of cochlear trauma. Odds ratio (95%CI) = 0.73, favoring no electrode type over the other.

## Discussion

The arrays currently available are straight and precurved, which include the perimodiolar and midscala electrodes. Translocating perimodiolar electrodes to the scala vestibuli from the scala tympani is more common due to the stiffness and rigidity of the electrode. Midscala electrodes were introduced because they are less rigid and less traumatic and they can be placed in the scala tympani without contact with lateral wall or the modiolus. Shorter lateral wall arrays yield the best results in terms of residual hearing preservation, but since they are near to the lateral wall of the cochlea, they are more distant from ganglion cells for stimulation than the precurved electrodes. 2 26 The present study was conducted to compare different electrodes in terms of trauma to the cochlea and hearing preservation. To best of our knowledge, the current is the largest meta-analysis in the literature about electrode types.

In the present study, the rates of hearing preservation were higher for the lateral wall (53%) and midscale (58%) electrodes compared with the perimodiolar electrode (35%).

Harrison et al. 12 conducted a study using lateral wall and perimodiolar electrodes, and they found no statistically significant difference between the electrodes in terms of preservation of residual hearing (mean rate: 52.9%). Mady et al. 16 conducted a study on 45 patients using the lateral wall and perimodiolar electrodes, and they found that lateral wall arrays yielded significantly better residual hearing preservation than perimodiolar electrodes.

The outcomes of residual hearing preservation in the literature differ widely, due to differences in reporting methods in different populations. Residual hearing preservation results in better speech scores and recognition in noise, better use of EAS, and subjective improvement in sound quality. 13 27

In the current study, the rates of cochlear trauma were as follows: lateral wall – 61%; perimodiolar – 29%; and midscala – 16%.

Boyer et al. 2 implanted 31 patients with perimodiolar arrays and 30 with lateral wall arrays, and they found 26% of scalar dislocation for perimodiolar electrodes compared with 3% for lateral wall electrodes. Precurved electrodes are stiffer than the straight ones, so they have more frictional forces on cochlear structures at their insertion. Dees et al. 8 conducted a study on 14 patients with midscala electrodes, and only 1 electrode (7%) changed its position.

Durakovic et al. 9 found that 5 cases out of 76 (6.6%) presented scalar translocations with perimodialar electrodes, and 12 cases out of 61 (20%) had preserved residual hearing. Selleck et al. 18 found 82% of residual hearing preservation with lateral wall electrodes in a study with 45 patients.

Soda-Merhy et al. 20 found no statistically significant differences in preservation of residual hearing between straight and perimodiolar electrodes. Svrakic et al. 21 found that midscala electrodes yielded better preservation of residual hearing than lateral wall electrodes. This is in line with the findings made by Todt et al. 22 Wanna et al. 25 reported that perimodiolar electrodes yielded less hearing preservation than lateral wall and midscala electrodes, and that perimodiolar electrodes were better suited for the scala vestibuli than lateral wall electrodes.

## Final Comments

The rates of preservation of functional residual hearing in CI are higher for lateral wall and midscala electrodes compared to perimodiolar electrodes.
